# Laser induced crystallization of Co–Fe–B films

**DOI:** 10.1038/s41598-021-93009-x

**Published:** 2021-07-08

**Authors:** Maria Almeida, Apoorva Sharma, Patrick Matthes, Nicole Köhler, Sandra Busse, Matthias Müller, Olav Hellwig, Alexander Horn, Dietrich R. T. Zahn, Georgeta Salvan, Stefan E. Schulz

**Affiliations:** 1 Center for Microtechnologies, Chemnitz University of Technology, 09126 Chemnitz, Germany; 2 Institute of Physics, Chemnitz University of Technology, 09126 Chemnitz, Germany; 3 Fraunhofer-Institute for Electronic Nano Systems, 09126 Chemnitz, Germany; 4 Laser Institut Hochschule Mittweida, University of Applied Sciences, 09648 Mittweida, Germany; 5 Institute of Ion Beam Physics and Materials Research, Helmholtz-Zentrum Dresden-Rossendorf, 01328 Dresden, Germany; 6 Center for Materials, Architectures and Integration of Nanomembranes, Chemnitz University of Technology, 09126 Chemnitz, Germany

**Keywords:** Electronic and spintronic devices, Magnetic properties and materials, Spintronics

## Abstract

Local crystallization of ferromagnetic layers is crucial in the successful realization of miniaturized tunneling magnetoresistance (TMR) devices. In the case of Co–Fe–B TMR devices, used most successfully so far in applications and devices, Co–Fe–B layers are initially deposited in an amorphous state and annealed post-deposition to induce crystallization in Co–Fe, thereby increasing the device performance. In this work, first direct proof of locally triggered crystallization of 10 nm thick Co–Fe–B films by laser irradiation is provided by means of X-ray diffraction (XRD) using synchrotron radiation. A comparison with furnace annealing is performed for benchmarking purposes, covering different annealing parameters, including temperature and duration in the case of furnace annealing, as well as laser intensity and scanning speed for the laser annealing. Films of Co–Fe–B with different stoichiometry sandwiched between a Ru and a Ta or MgO layer were systematically assessed by XRD and SQUID magnetometry in order to elucidate the crystallization mechanisms. The transformation of Co–Fe–B films from amorphous to crystalline is revealed by the presence of pronounced CoFe(110) and/or CoFe(200) reflexes in the XRD θ-2θ scans, depending on the capping layer. For a certain window of parameters, comparable crystallization yields are obtained with furnace and laser annealing. Samples with an MgO capping layer required a slightly lower laser intensity to achieve equivalent Co–Fe crystallization yields, highlighting the potential of laser annealing to locally enhance the TMR ratio.

## Introduction

In the past years, Co–Fe–B alloys have found a wide range of applications in spintronic devices, such as for biomedical sensing platforms, magnetic recording, and communication applications^1–4^. In particular, these alloys have proven to be fundamental in the development of magnetic tunnel junctions (MTJs), providing large tunneling magnetoresistance (TMR) ratios using an MgO barrier. Following predictions of large TMR yields on Fe/MgO/Fe layers^5,6^, the poor lattice matching of the body-centered cubic (bcc) Fe(-Co) phase on well oriented (001) MgO presented a great constraint towards the realization of devices^7,8^. A practical solution was found in Co–Fe–B as an alternative electrode material^9^, which provides a smooth amorphous template for the growth of MgO with the required (001) crystalline orientation. Post-deposition annealing induces the formation of a bcc Co–Fe phase imprinted by MgO, resulting in high TMR yields^10^.

In the context of TMR devices, this annealing step is typically employed not only to induce the Co–Fe–B crystallization but also to pin the magnetization of one of the ferromagnetic layers via the exchange bias effect with an additional antiferromagnetic layer^11^. The latter introduces an upper limit in terms of the annealing temperature, mainly due to diffusion processes, leading to degradation of the magnetic properties of the layers and TMR yields^12,13^. Laser irradiation has proven to be an efficient alternative to set the exchange bias locally^14^, allowing the realization of sensors with a multidimensional spatial resolution^15^. The challenge in employing this method in MTJs lies in the fact that the annealing ideally should also induce the crystallization requirements to achieve large TMR ratios. Although laser-assisted crystallization has been investigated in ferromagnetic amorphous ribbons^16,17^, to this date no systematic study has addressed Co–Fe–B sputtered thin films for TMR applications and the implications of the choice of the seed and capping layer materials on this crystallization process.

In the present work, first direct proof of the locally triggered crystallization of Co–Fe–B thin films is provided by X-ray diffraction (XRD) measurements performed with synchrotron radiation at BESSY II endstation KMC-2. A systematic investigation of the crystallization of Co–Fe–B with different Co–Fe compositions and Ta or MgO capping layers was performed, with a detailed variation of different laser annealing parameters and a comparison to vacuum furnace annealing. Focus was given to simple layer stacks, in order to gain deeper insight into the changes in crystal structure of Co–Fe–B depending on neighboring layers typically found in practical TMR devices for applications. The non-destructive nature of XRD measurements and flexibility in measurement geometries present at KMC-2 enabled a detailed comparison over a wide range of oven and laser annealing parameters. This allowed not only a range of laser intensities and laser scanning speeds to be determined, where comparable levels of crystallization of Co–Fe–B are achieved, but also to identify differences arising from a dynamic heating process with inherently large thermal gradients as common for fast, localized laser annealing, thus paving the way for better and more flexible future applications with TMR devices.

## Results

**Figure 1:**
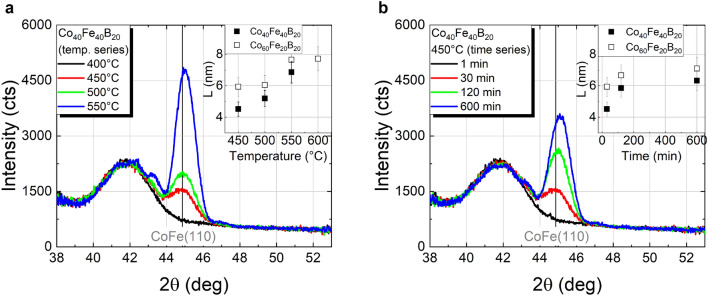
XRD *θ*-2*θ* scans of Co_40_Fe_40_B_20_ with a Ta capping layer furnace annealed for 30 min at temperatures in the range from 400 °C to 550 °C (**a**), and at 450 °C for different annealing durations (**b**). The vertical coherence lengths determined from the CoFe(110) reflex for both Co–Fe compositions are shown in the insets.

XRD *θ*–2*θ* scans of Co_40_Fe_40_B_20_ grown on a Ta/Ru seed layer and capped with Ta furnace annealed in vacuum for 30 min at different temperatures (temperature series), and at 450 °C with different annealing time (time series) are shown in Fig. 1. The pronounced Co–Fe (110) reflex around 2*θ* ≈ 44.8° detected after annealing for 30 min at 450 °C and above (in terms of both, annealing time and temperature) indicates the crystallization of the Co–Fe–B films into body-centered cubic (bcc) Co–Fe, as expected upon the migration of B out of the Co–Fe lattice, for instance to grain boundaries^18,19^. A further less intense reflex corresponding to Co–Fe (220) is also measured at 2*θ* ≈ 88°, and the lack of additional reflexes of bcc Co–Fe suggests crystallization of the film with a strong (110) texture, as further discussed below. A visible improvement of the crystallinity is observed with increasing temperature and time, whereas no significant changes of the broad peak are found at 2*θ* ≈ 42°, related to the hexagonal close-packed (hcp) Ru(002) seed layer. At a closer inspection, satellite peaks are visible on both sides of the CoFe(110) Bragg peak that can be assigned to Laue oscillations of the Co–Fe reflex due to very smooth interfaces. The obtained peak period matches the 10 nm Co–Fe–B film thickness. Similar results were found by replacing Co_40_Fe_40_B_20_ with Co_60_Fe_20_B_20_ (shown in Fig. S1 of supplementary information), with the main difference related to a more intense Co–Fe peak, in good agreement with previous studies on thicker films of these two compositions^19^. A single disparity is found between the results of 10 nm thin films and those previously reported for 100 nm thick films: whereas the crystallization of Co–Fe–B in the present work features a well-defined CoFe(110) texture for both compositions, 100 nm thick Co–Fe–B films in the previous study crystallized into polycrystalline Co_50_Fe_50_ (from amorphous Co_40_Fe_40_B_20_) and into well (110) textured Co_75_Fe_25_ (from amorphous Co_60_Fe_20_B_20_). This discrepancy in the crystallization of Co_40_Fe_40_B_20_ in both studies is most likely due to the different cap layers (since it was shown that a Pt cap layer promotes the nucleation centers for crystallization from the top), and possibly due to significant differences in the investigated layer thicknesses.

The vertical coherence lengths (*L*), as a minimum estimate of the crystallite sizes in the normal direction to the sample surface, were calculated from the full width at half maximum (*FWHM*_*2θ*_) of the CoFe(110) reflex using the Scherrer equation^20^:

1 $$\text{L}=\frac{\lambda \cdot \text{K}}{{\text{FWHM}}_{{2 \theta}} \cdot \cos( \theta )}$$

where *λ* = 0.15406 nm was chosen throughout our experiments in accordance to Cu-K_α_ radiation, *θ* is the Bragg angle and *K* = 0.9 was chosen as shape factor, related to cubic crystallites. The dependence of *L* on the annealing temperature and annealing duration is shown in the insets of Fig. 1. The largest crystallites are found after furnace annealing for 30 min at 550 °C, with a vertical coherence length in the order of ~ 7 nm for Co_40_Fe_40_B_20_. The apparent saturation for larger temperatures or longer annealing likely indicates a complete crystallization of the layer, especially considering a nominal Co–Fe–B film thickness of 10 nm. The temporal evolution in the case of the time dependent annealing at 450 °C (*cf.* inset of Fig. 1b) may arguably be in good agreement with the isothermal growth kinetics formalism provided by Johnson, Mehl, Avrami, and Kolmogorov (JMAK model)^21^. However, given the reduced amount of experimental data points, a detailed analysis for a systematic evaluation of the crystallization under this model is not reasonable and is outside the scope of this work. In terms of both Co–Fe compositions, although the same trends are found for both annealing series, *L* was observed to be approximately 0.9 nm larger for Co_60_Fe_20_B_20_. This difference is in good agreement with the thicknesses obtained from the Laue oscillations analysis: ~ 9.9 nm for Co_40_Fe_40_B_20_ and ~ 10.9 nm Co_60_Fe_20_B_20_ corresponding to the actual film thicknesses.

**Figure 2:**
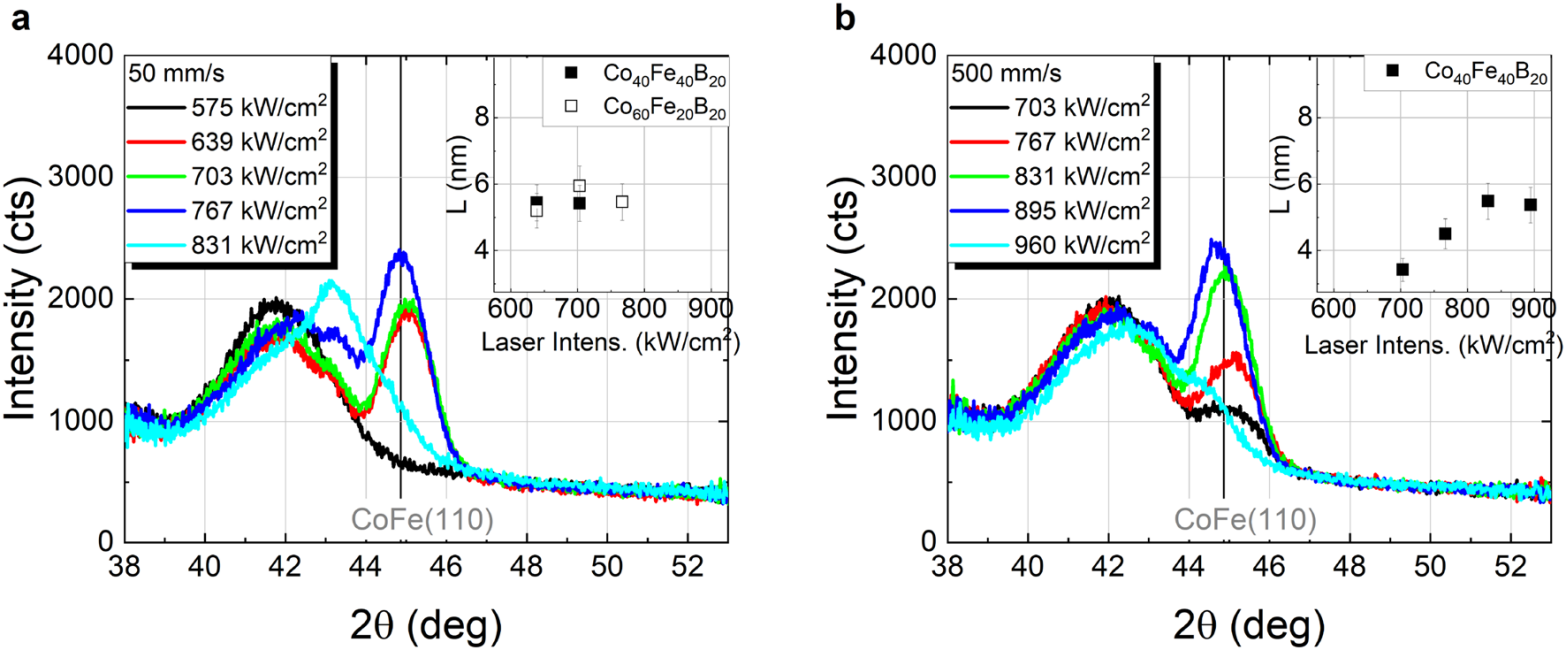
XRD *θ*-2*θ* scans of Co_40_Fe_40_B_20_ annealed by cw laser irradiation in dependence of laser intensity at different scanning speeds: (**a**) 50 mm/s and (**b**) 500 mm/s. The vertical coherence lengths (*L*) determined from the CoFe(110) peak are shown in the insets.

The *θ*–2*θ* scans of Co_40_Fe_40_B_20_ capped with a Ta layer locally annealed with continuous wave (cw) laser radiation are shown in Fig. 2, for 50 mm/s and 500 mm/s scanning speeds (further scans for 5000 mm/s are shown in Fig. S2 of the supplementary information). The CoFe(110) reflex is observed for all investigated scanning speeds in the range of laser intensities summarized in Table 1. Consistent results were found for Co_60_Fe_20_B_20_ at 50 mm/s, although only this particular scanning speed was tested over the course of this study (*θ*–2*θ* scans for this sample are shown in Fig. S3 of the supplementary information). Within the depicted range of laser intensities, there are some differences with respect to the furnace annealed samples. Whereas for furnace annealing an increase in temperature or annealing time leads to no significant changes of the Ru(002) and a slight shift of the CoFe(110) reflex toward higher angles, increasing laser intensities result in Ru(002) reflex displacement toward higher angles accompanied by a slight decrease of its intensity, as well as a shift of CoFe(110) toward lower angles. Beyond the upper limit of these ranges in case of laser annealing, the CoFe(110) reflex is no longer clearly observed, possibly as a result of alloying with Ta^22^ and Ru from the seed layer by laser irradiation. In fact, previous X-ray photoemission depth profiling studies demonstrated significant diffusion of Ru and Co for laser intensities beyond 900 kW/cm^2^ for 50 mm/s and 500 mm/s scanning speeds^23^.

**Table 1:** Range of laser intensity values inducing Co–Fe crystallization as proven by XRD *θ*-2*θ* scans of Co_40_Fe_40_B_20_ capped with Ta or MgO/Ta; dwell time according to each laser scanning speed.

Scanning speed (mm/s)	Range of laser intensity, which can be used to obtain films exhibiting a CoFe(110) or CoFe(200) reflex		Dwell time, *τ* (µs)
	Co_40_Fe_40_B_20_/Ta	Co_40_Fe_40_B_20_/MgO/Ta	
50	640–770 kW/cm^2^	< 575–770 kW/cm^2^	400
500	< 700–900 kW/cm^2^	< 700–830 kW/cm^2^	40
5000	960– > 1020 kW/cm^2^	–	4

The vertical coherence lengths, obtained with Eq. (1) from the CoFe(110) reflex, reveal a maximum crystallite size of approximately 5.5 nm for Co_40_Fe_40_B_20_ upon annealing with cw laser radiation at 50 mm/s and 500 mm/s scanning speeds, or up to 4.5 nm at 5000 mm/s (*cf.* insets of Fig. 2 and Fig. S2 of supplementary information). Compared to vacuum furnace annealed samples, the maximum levels of crystallization are similar to those achieved at temperatures in the range of 450 °C to 500 °C for 30 min furnace annealing, which is remarkable given the characteristic time scales of the laser irradiation processes. These are defined by dwell times (*τ* = beam diameter/scanning speed, cf. Table 1) well below the millisecond range, proving the potential of laser annealing for fast crystallization of complex ultra-thin film layer stacks. It should be mentioned that in isothermal annealing studies of Co–Fe–B time intervals in the range of seconds were required for the crystallization onset. For example, the incubation time, *i.e.* the time interval until the crystalline grains start to grow, showed an inversely linear behavior with the annealing temperature^21^, with a value of 6.2 s at 460 °C^24^. Even though the crystallization kinetics may be considerably different in the case of the laser annealing due to its non-isothermal characteristics, the significantly shorter time scales suggest that significantly higher temperatures are required in order to induce the crystallization of Co–Fe–B. This interplay between the short time scales and the energy provided to the system by the laser radiation is likely to constitute the major limiting factor to the formation of larger crystallites, as too large laser intensities may cause alloying with adjacent layers. This would further explain the tendency of a decreasing vertical coherence length *L* with the laser intensity (see insets of Fig. 2 and Fig. S2 of the supplementary information) prior to the merge of the CoFe(110) and Ru(002) peaks.

**Figure 3:**
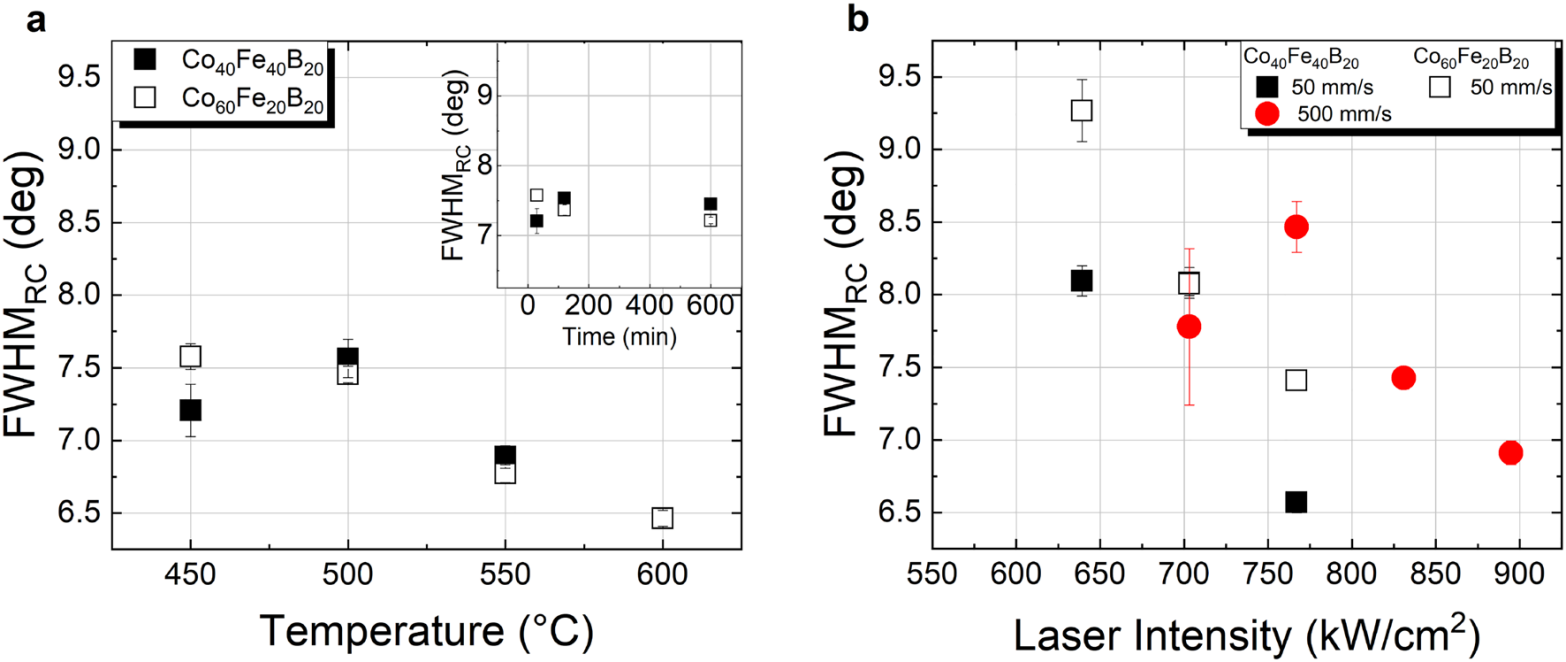
Comparison of the rocking curve FWHM for both Co–Fe compositions with a Ta capping layer annealed: (**a**) in furnace for 30 min at temperatures in the range of 450 °C–600 °C and at 450 °C at different times (inset); (**b**) by cw laser irradiation with different scanning speeds and different laser intensities (please note the overlap of data points for Co_40_Fe_40_B_20_ (filled squares) and Co_60_Fe_20_B_20_ (empty squares) at 700 kW/cm^2^ laser intensity, 50 mm/s scanning speed).

The Co–Fe thin films mosaicity was further investigated by a rocking-scan analysis of the CoFe(110) crystallites, confirming the formation of a well-defined (110) texture, too, see Fig. 3. The decrease of the rocking curve FWHM (FWHM_RC_) with increasing furnace annealing temperature is consistent with an improvement in the crystallite orientation (Fig. 3a). Similarly, with laser annealing, an improvement of the (110) texture with increasing intensity is observed for 50 mm/s and 500 mm/s (cf. Fig. 3b). Since for 5000 mm/s the limited range of tested intensities and low crystallization yields hindered a more detailed analysis with no pronounced peaks below 1000 kW/cm^2^, the results are not shown. Even though the degree of structural ordering, *i.e.* of the crystalline volume, increases with longer annealing duration, this seems to have less or no effect on the crystal quality regarding crystallographic misalignments, as shown in the inset of Fig. 3a. Therefore, the mosaicity is defined mostly by the annealing temperature, or in the case of laser annealing, by the laser intensity.

**Figure 4:**
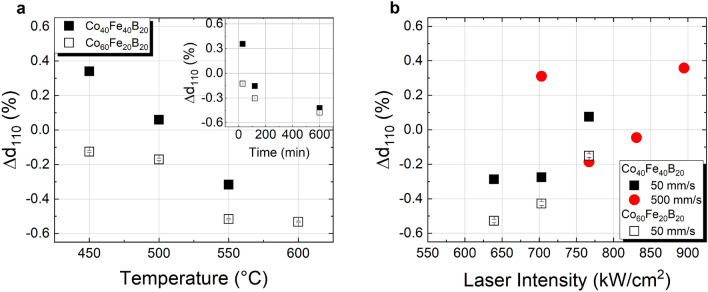
Comparison of CoFe(110) d-spacing deviation from database values for Co_40_Fe_40_B_20_ and Co_60_Fe_20_B_20_ (both with Ta cap) annealed (**a**) in the furnace for 30 min at temperatures of 400 °C–600 °C and at 450 °C at different times (inset), and (**b**) annealed by cw laser irradiation at different scanning speeds and laser intensities.

The spacing of the CoFe(110) lattice planes parallel to the sample surface, *d*_110,⊥_, was evaluated by comparing the experimental values obtained from the CoFe(110) reflex against reported literature values, taking into account the respective Co–Fe composition^25,26^, see Fig. 4. In the case of the samples annealed in furnace, both, increasing temperature and annealing time lead to a decrease of *d*_110,⊥_ to values below the literature values. In fact, additional in-plane XRD measurements of the CoFe(110) reflex show a 0.4% larger spacing of lattice planes perpendicular to the sample surface, *d*_110,||_, for Co_40_Fe_40_B_20_ (Ta cap) than the reported literature values^25^, with negligible variances upon increase of furnace temperature (depicted in Fig. S4a of supplementary information). In summary, this corresponds to a contraction of the CoFe(110) planes in the <001> direction to compensate the expansion in the <110> direction arising at the Ru_hcp_/CoFe_bcc_ interface. This is in line with the bcc-hcp orientation relationship described by the Burgers mechanism^27,28^ and may correspond to the formation of a pseudomorphic layer at that interface, as was previously reported for Fe_bcc_/Ru_hcp_^29^.

For the laser annealing, while a similar trend of decreasing out-of-plane lattice spacing with increasing laser intensity can be observed in exceptional cases only (for 500 mm/s up to 760 kW/cm^2^ and for 5000 mm/s—not shown), the general trend is an increase in lattice spacing with increasing laser intensity, see Fig. 4b. The in-plane XRD measurements of those samples locally annealed by cw radiation at 50 mm/s scanning velocity further reveal an increase of the *d*_(110)_ lattice spacing of planes perpendicular to the sample surface, too, indicating an enlargement of the lattice in both, horizontal and vertical direction with increasing laser intensity (see Fig. S4b in Supplementary Information). This suggests that, even though increasing laser intensity may be beneficial in terms of the crystalline order as observed by means of the rocking curve analysis, additional stress in the lattice or other effects, such as modulation at the CoFeB/Ta^30^, may occur, most likely due to the significant temperature gradients inherent to the process.

**Figure 5:**
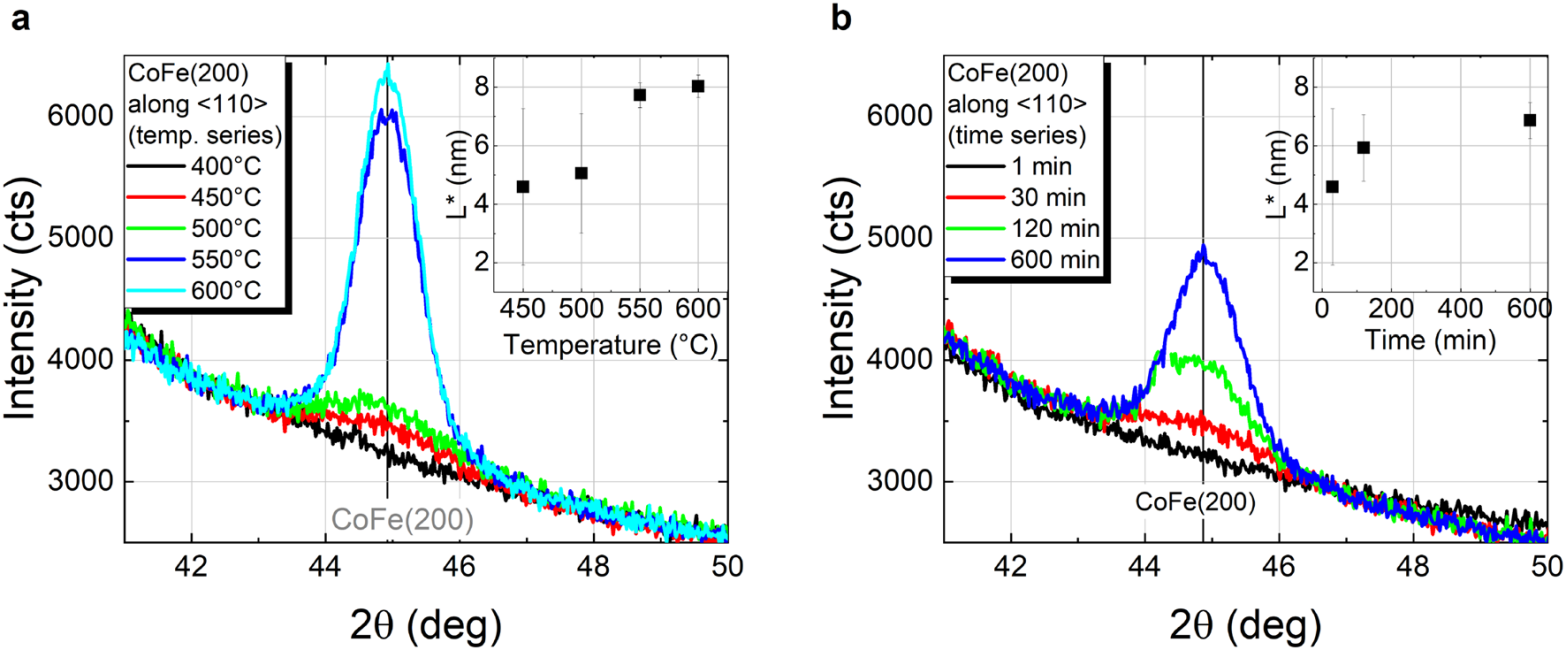
XRD off-specular θ–2θ scans (sample tilt of *χ* = 45°) measuring the (110) peak of CoFe(200) crystallites of Co_40_Fe_40_B_20_ capped with MgO annealed in the furnace for (**a**) 30 min at temperatures in the range 450 °C–600 °C and (**b**) different durations at a constant annealing temperature of 450 °C. Coherence lengths along the <110> direction, L*, in dependence of the respective annealing temperature/time are plotted in the insets.

In the case of Co_40_Fe_40_B_20_ grown on Ta/Ru and with an MgO/Ta capping layer, the MgO layer with (100) orientation acts as a template for the crystallization of Co–Fe–B into Co–Fe with distinct (100) texture, as expected^31,32^. This is verified by the observation of a CoFe(200) reflex in the *θ*-2*θ* scan and the absence of reflexes related to other crystallographic orientations of bcc Co–Fe, mainly the CoFe(110) previously observed for Ta capped samples (see Fig. S5 in supplementary information). In order to circumvent the influence of the dominating Si substrate peak at 2*θ* = 69.2° on the CoFe(200) reflex at 2*θ* = 65.3°, off-specular *θ*-2*θ* measurements of the CoFe <110> direction were performed (sample tilted by *χ* = 45°). In this configuration, first signs of crystallization are found after furnace annealing for 30 min at 450 °C, with a peak evolution with temperature/time in good agreement with that of Co_40_Fe_40_B_20_ capped with Ta, being (110) textured, see Fig. 5. In the standard configuration (*χ* = 0°) the CoFe(200) reflex can only be clearly analyzed for furnace annealing at 550 °C and 600 °C.

A coherence length value determined from the CoFe(110) reflex along the <110> direction, L*, was further calculated using expression (1) and is shown in the insets of Fig. 5. Please note that due to the differences in the measured geometries (*χ* = 0° and *χ* = 45°, respectively), a comparison to the values of *L*, previously calculated for Co_40_Fe_40_B_20_ capped with Ta, solely focuses on evaluating the trends in dependence on annealing parameters. In fact, the increase of L* with annealing temperature and duration is remarkably similar to that observed in Fig. 1 for samples capped with Ta. In this case, the maximum coherence length values obtained are ~ 8 nm for 30 min annealing above 550 °C, and ~ 7 nm after annealing at 450 °C for 600 min.

**Figure 6:**
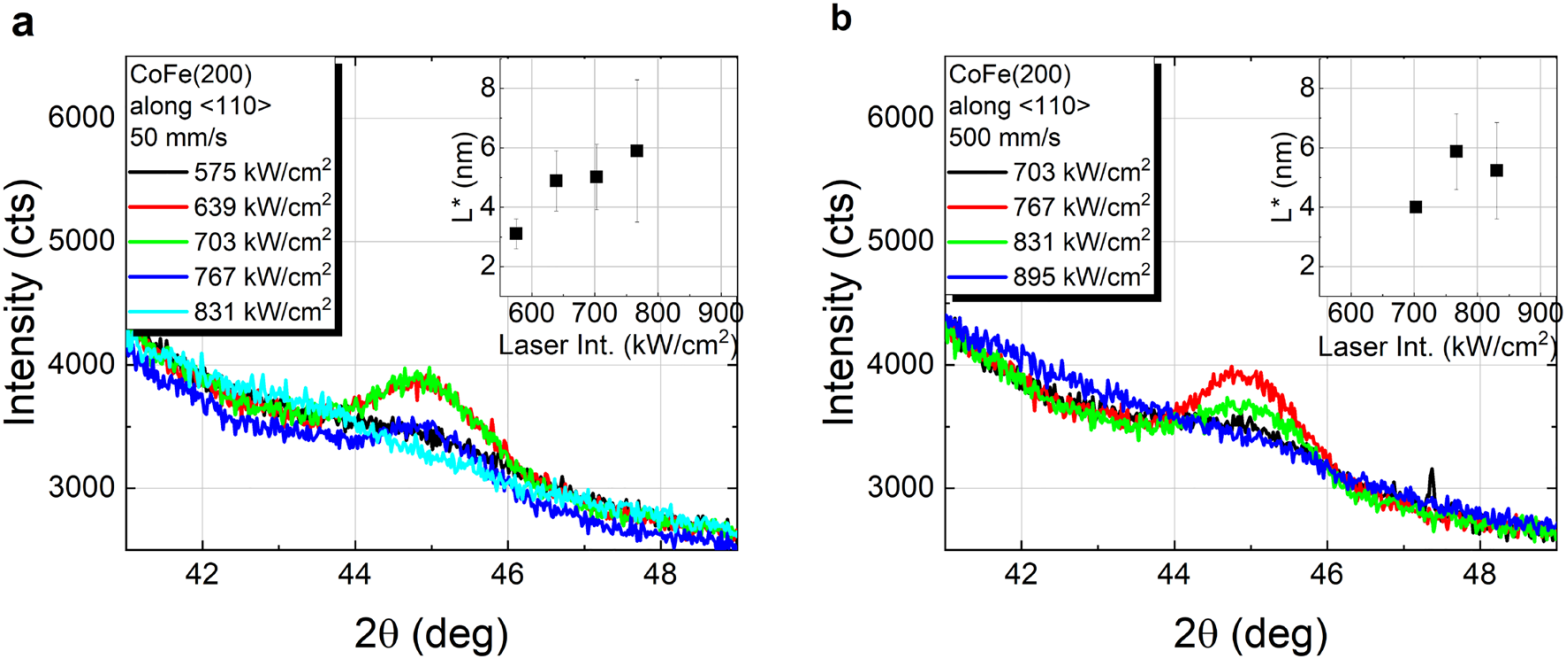
XRD off-specular θ–2θ scans (sample tilt of *χ* = 45°) measuring the (110) peak of CoFe(200) crystallites of Co_40_Fe_40_B_20_ capped with MgO annealed by laser irradiation at (**a**) 50 mm/s and (**b**) 500 mm/s scanning speed. Coherence lengths along the <110> direction, L*, in dependence of the laser intensities are plotted in the insets.

In the case of the laser annealing, besides serving as a protective layer for the MgO (preventing water adsorption), the Ta limits the amount of light contributing to the crystallization process due to its high reflectance. However, a Ta layer was used on MgO too, in order to allow for a better comparison of the irradiation parameters to those previously discussed on the samples with a single Ta capping layer. In this case, even though the trend of L* resembles that of *L* of Co_40_Fe_40_B_20_ capped with only Ta, a slight variation can be found in terms of the laser intensity ranges, where crystallization of CoFe occurs, see Fig. 6 and Table 1. In the case of laser annealing at 50 mm/s scanning speed, first appearance of the CoFe(200) peak is observed at around 575 kW/cm^2^, resulting in a broader range of laser intensities, where crystallization can be achieved, compared to the sample capped only with Ta. At 500 mm/s, even though no laser intensities below 700 kW/cm^2^ were investigated, the onset for alloying/diffusion decreased significantly in comparison to that of a single Ta capping layer. For those samples, a CoFe(110) reflex was observable up to 960 kW/cm^2^, whereas in the case of an MgO/Ta capping layer, at 900 kW/cm^2^ the CoFe(110) reflex along the <110> direction was no longer found, inferring a lower laser intensity threshold, above which layer deterioration is observed. This implies that the MgO cap influences significantly the heat distribution in the Co–Fe–B layer in the case of laser annealing. Given the upper Ta layer on both samples and the transparency of MgO at the used laser wavelength (*λ* = 1064 nm), the amount of energy provided by the laser irradiation should be comparable for both samples. The main difference lies therefore, in the thermal properties of MgO, which has a lower thermal conductivity than Ta^33,34^. In particular, in the case of localized laser annealing the thermal conductivity of the cap layer plays a crucial role in the lateral heat dissipation through the cap layer. By decreasing the heat diffusion from the Co–Fe–B layer, the MgO layer retains more thermal energy that contributes to the crystallization process, hence requiring lower laser intensities to achieve the same yields. This is particularly interesting from the point of view of future applications, since the use of such transparent materials can play a key role in finding optimum laser parameters for the laser annealing, where crystallization is maximized with minimum diffusion of species across the layer stack. In fact, the applied laser annealing offers crystallization yields of Co_40_Fe_40_B_20_ to CoFe(200) (similar to CoFe(110) as shown before), well comparable to those obtained with furnace annealing at temperatures in the range of 450 °C-500 °C for 30 min, revealing a great potential for TMR applications.

**Figure 7:**
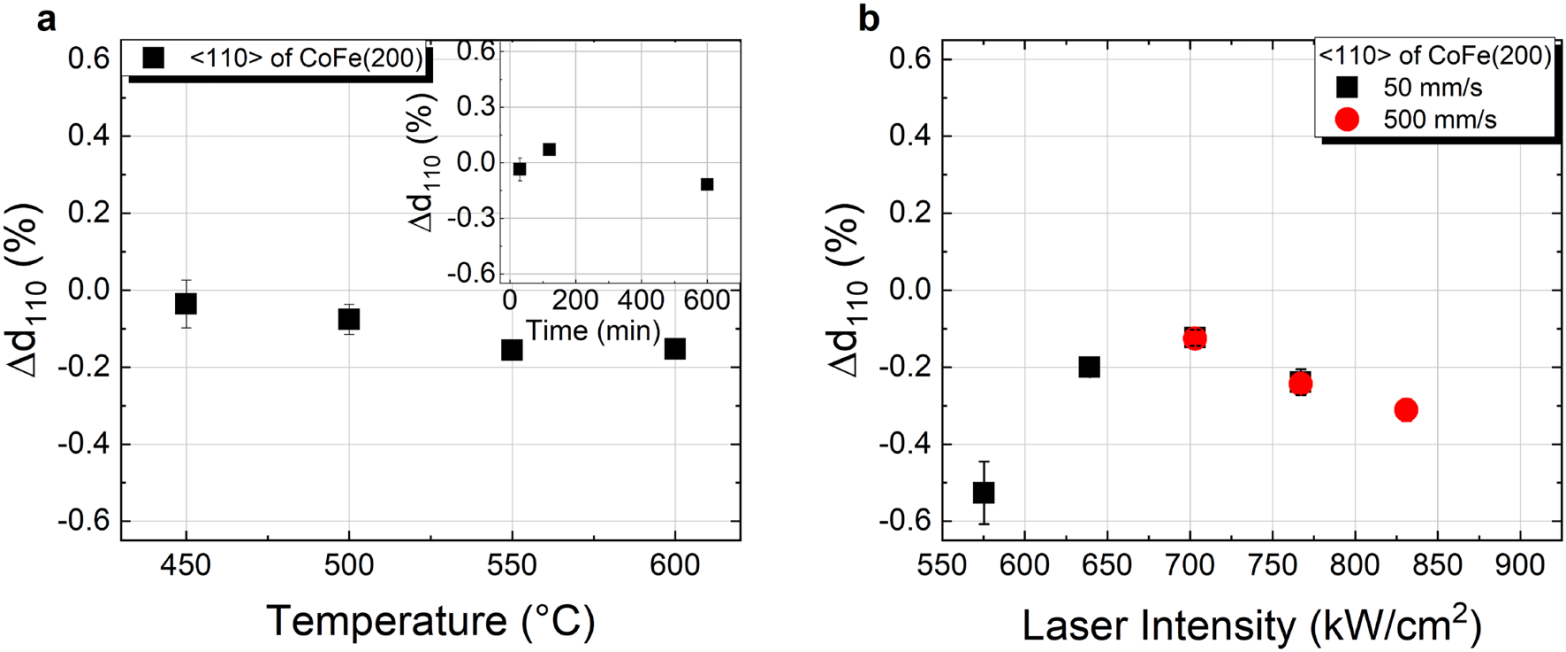
Deviation of *d*-spacing of CoFe(110) from the database value of Co–Fe for Co_40_Fe_40_B_20_ capped with MgO/Ta annealed (**a**) in the furnace for 30 min at temperatures of 400 °C–600 °C and at 450 °C at different times (inset), and (**b**) annealed by cw laser irradiation at different scanning speed and laser intensity, calculated from the off-specular measurements (sample tilt of *χ* = 45°).

The *d*-spacing of Co–Fe planes along the <110> direction, *d*_110,<110>_, is in good agreement with reported literature values^25^, especially for the furnace annealing, as shown in Fig. 7. Since in this case the distance measured refers to planes at 45° to the sample surface, an immediate link to the lattice transformations shown previously for Co_40_Fe_40_B_20_ capped with Ta is not straightforward, yet allowing a qualitative comparison between furnace and laser annealed samples. Some considerations can be made regarding those few samples, where a CoFe(200) reflex is visible despite the dominating Si substrate peak, namely those annealed at 550 °C and 600 °C (shown in Fig. S5 of the supplementary information). For those samples, a decrease of ~ 0.8% in *d*_200,⊥_ is observed compared to the literature values^25^, corresponding to a compression of the CoFe(200) planes parallel to the sample surface. On the other hand, the respective CoFe(110) reflex observed by in-plane measurements shows an expansion in CoFe(110) planes perpendicular to the sample surface, in the order of ~ 0.7%. The latter is likely to arise from the lattice mismatch at the Co–Fe/MgO interface: whereas the (110) plane of Co–Fe aligns along the (100) plane of MgO, a mismatch of ~ 4.1% is expected^25,35^ and values of ~ 3% were reported previously^32^, due to a comparatively larger lattice of MgO. In the case of the laser annealed samples, although the, *d*_110,<110>_ shows slightly larger variations with laser intensity than furnace annealed samples, comparable values are found for the samples with best crystallization yields (*i.e*. approximately in the range 625 kW/cm^2^ to 775 kW/cm^2^, for 50 mm/s and 500 mm/s scanning speed). An evident difference was found for the *d*_110,||_ resulting from in-plane XRD measurements: whereas the (110) planes of Co–Fe perpendicular to the sample surface show, similarly to the furnace annealed samples, a larger spacing than that reported in the literature^25^, this deviation is even larger for laser annealed samples (*d*_110,||_> 1.3%). The lack of a distinguishable CoFe(200) reflex on the *θ*-2*θ* scans measured in standard out-of-plane configuration (*χ* = 0°) hinders a more detailed analysis but such an expansion of the lattice may relate to the lower thermal conductivity of MgO at the Co–Fe–B /MgO interface^33^.

**Figure 8:**
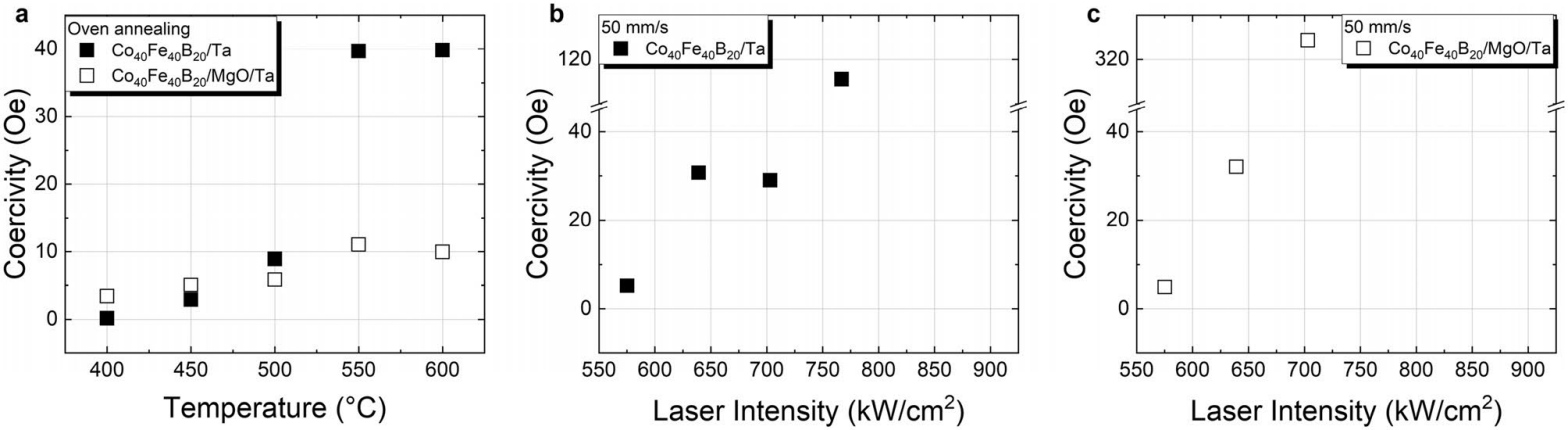
Coercive fields of Co_40_Fe_40_B_20_ capped with Ta or MgO/Ta determined by SQUID magnetometry measuring *M*(*H*) hysteresis loops up to magnetic saturation at room temperature for (**a**) samples annealed in furnace for 30 min at different temperatures; (**b**, **c**) annealed by cw laser irradiation in dependence of laser intensity at 50 mm/s.

In terms of the magnetic behavior, some significant differences arise on the coercivity of Co_40_Fe_40_B_20_ capped with Ta or MgO/Ta for furnace and cw laser annealing at 50 mm/s, as shown in Fig. 8. In furnace annealed samples, a slight increase of the coercivity with temperature is observed up to 500 °C, with comparable values for both capping layers. Above 500 °C a pronounced increase from ~ 10 Oe (at 500 °C) to ~ 40 Oe (at 550 °C) occurs for Co_40_Fe_40_B_20_ capped with Ta, which is not observed in the case of MgO capping. Although such an increase in coercivity could relate to the increase in crystallite size reflected by the vertical coherence length shown previously (Fig. 1a), the fact that this is not observed for Co_40_Fe_40_B_20_ capped with MgO/Ta suggests that crystallization alone may not be the only factor contributing to this increase in coercivity. In this way, the significant lattice changes observed in the case of the Ta capping (Fig. 4a) are likely to be more relevant, since such a distortion of the lattice due to the Ru_hcp_/Co–Fe_bcc_ interface could hinder the domain wall motion through defects, resulting in an increase of coercivity. In fact, such an interplay between the changes in the lattice and coercivity of Co–Fe could explain the results on laser annealed samples, too, see Fig. 8b and c. The systematic increase of coercivity with laser intensity of Co_40_Fe_40_B_20_ capped with Ta and MgO/Ta annealed at 50 mm/s scanning speed are in line with the observed expansion of the lattice, particularly in-plane. The dramatic increase in coercivity to 115 Oe at 780 kW/cm^2^, in the case of the Ta capping, or even 325 Oe at 700 kW/cm^2^ in the case of the MgO/Ta capping layer are evidence for significant changes in the layers and interfaces, establishing an upper limit to the laser intensity. Below those intensities, coercive field values comparable to those of furnace annealed samples are obtained, which, along with the observed crystallization yields, further proves the potentiality of laser annealing for TMR devices.

## Conclusions

In this work, the locally triggered crystallization of Co–Fe–B thin film systems using cw laser irradiation was demonstrated for the first time. A detailed comparison between conventional furnace annealing and the proposed laser based method allowed a window of parameters to be identified, for which similar crystallization yields are observed. The observed dependence of the crystalline structure on the laser intensity and scanning speed emphasizes the dynamic nature of the process, punctuated by the fact that crystallization is achieved in much shorter time scales than those previously observed in isothermal studies. As further shown, the crystalline orientation of the adjacent layers is crucial to establish the crystallization of Co–Fe–B into CoFe(110) or CoFe(001), for furnace as well as for laser annealing. However, due to the local nature of the laser irradiation process, the thermal conductivity and the particular layer stack play, as well, a significant role, further influencing the window of parameters, in which crystallization of Co–Fe–B is observed. This interplay between laser energy absorption and the local heat distribution due to the characteristics of the adjacent layers is moreover observable in slight changes of the Co–Fe lattice parameters.

## Experimental methods

An automated Singulus Rotaris UHV sputtering system was used to deposit the following stacks at room temperature on thermally oxidized Si substrates:

A. Si/SiO_2_ (100 nm)/Ta (5 nm)/Ru (3 nm)/Co_40_Fe_40_B_20_ (10 nm)/Ta (5 nm)
B. Si/SiO_2_ (100 nm)/Ta (5 nm)/Ru (3 nm)/Co_60_Fe_20_B_20_ (10 nm)/Ta (5 nm)
C. Si/SiO_2_ (100 nm)/Ta (5 nm)/Ru (3 nm)/Co_40_Fe_40_B_20_ (10 nm)/MgO (10 nm)/Ta (5 nm)

The 200 mm wafers were diced into samples with a size of (6 × 6) mm^2^, which were further annealed using standard vacuum annealing, including a temperature series at fixed annealing duration (30 min at temperatures ranging from 400 °C to 600 °C) and a time series at a fixed temperature (1 min to 600 min at 450 °C). For the laser annealing experiments a Nd:YAG laser (1064 nm wavelength) with 10 µm focal radius was used in continuous wave (cw) mode at various laser intensities (120 kW/cm^2^ up to 1020 kW/cm^2^) and using different scanning speeds (50 mm/s; 500 mm/s and 5000 mm/s). Further details regarding this set-up can be found elsewhere^36^.

X-ray diffraction (XRD) was conducted at the KMC-2 endstation of the electron storage ring BESSY II of the Helmholtz-Zentrum Berlin für Materialien und Energie^37^. XRD was performed in *θ*-2*θ* geometry to probe the crystallization and crystallite size of the thin film samples. Furthermore, off-specular measurements and rocking curves were recorded to observe further Bragg peaks or evaluate the tilting of the crystallites. The vertical coherence length (*L*) was calculated using the Scherrer formula^20^. Additional in-plane XRD measurements were conducted using a SmartLab diffactometer from Rigaku, equipped with a rotating Cu anode operated at 9 kW.

The magnetic characterization was performed by superconducting quantum interference device-vibrating sample magnetometry (SQUID-VSM, by Quantum Design).

## Supplementary Information

Supplementary Information.
