# Supplementary material for: Laser induced crystallization of Co–Fe–B films
Source: Sci Rep. 2021 Jul 8;11:14104. doi: 10.1038/s41598-021-93009-x (PMC8266803; doi:10.1038/s41598-021-93009-x)
Supplement: Supplementary file 1 — Supplementary Information. [file 41598_2021_93009_MOESM1_ESM.docx]

**Supplementary information: Laser crystallization of Co-Fe-B films for TMR devices**

Maria Almeida^1,6,*^, Apoorva Sharma^2^, Patrick Matthes^3^, Nicole Köhler^1^, Sandra Busse^4^, Matthias Müller^4^, Olav Hellwig^5,6^, Alexander Horn^4^, Dietrich R.T. Zahn^2,6^, Georgeta Salvan^2,6^, and Stefan E. Schulz^1,3,6^

*^1^Center for Microtechnologies, Chemnitz University of Technology, 09126 Chemnitz, Germany*

*^2^Institute of Physics, Chemnitz University of Technology, 09126 Chemnitz, Germany*

*^3^Fraunhofer Institute for Electronic Nanosystems, 09126 Chemnitz, Germany*

*^4^Laser Institute, University of Applied Sciences, 09648 Mittweida, Germany,*

*^5^Institute of Ion Beam Physics and Materials Research, Helmholtz-Zentrum Dresden-Rossendorf, 01328 Dresden, Germany*

*^6^Chemnitz University of Technology, Center for Materials, Architectures and Integration of Nanomembranes, 09126 Chemnitz, Germany*

| 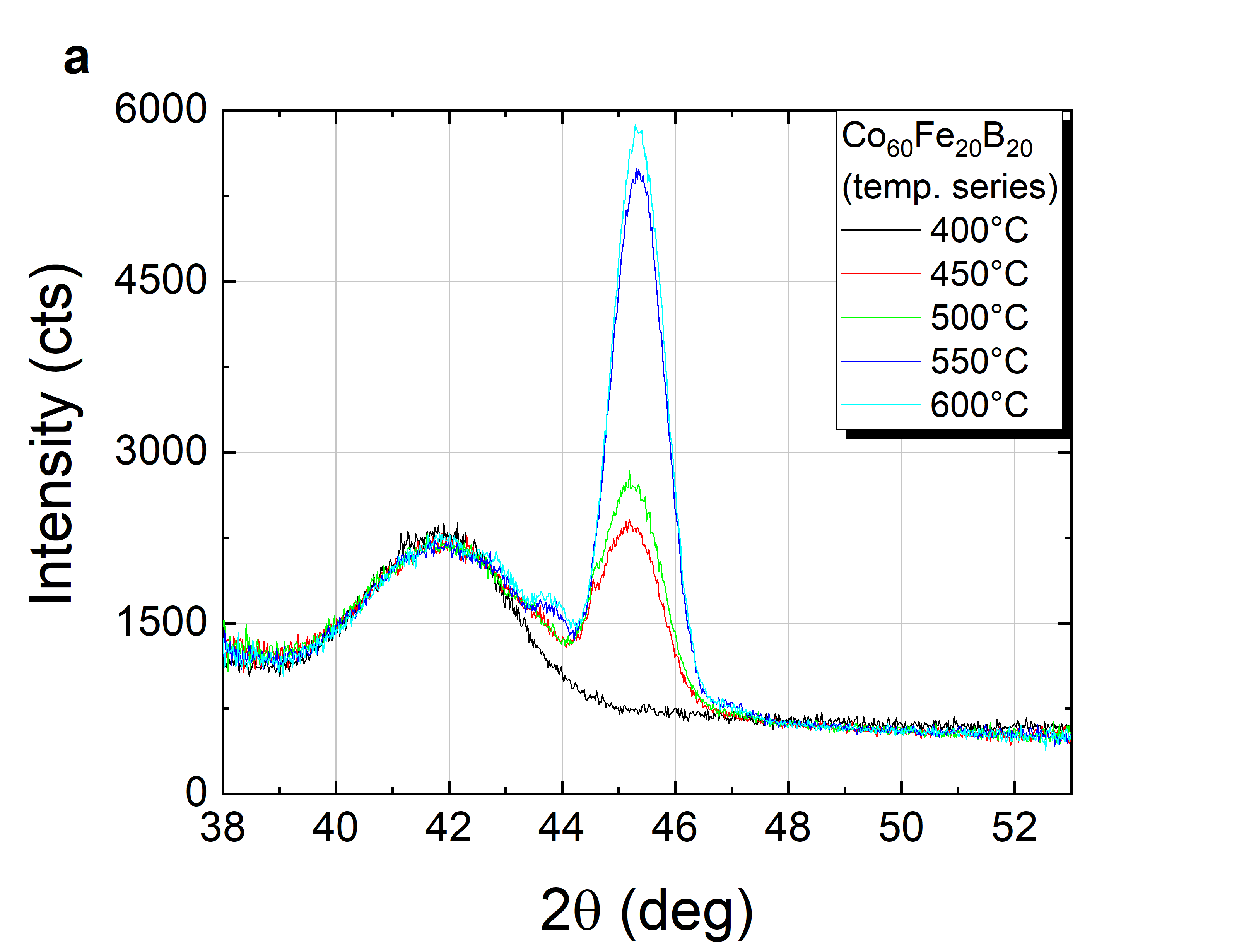 | 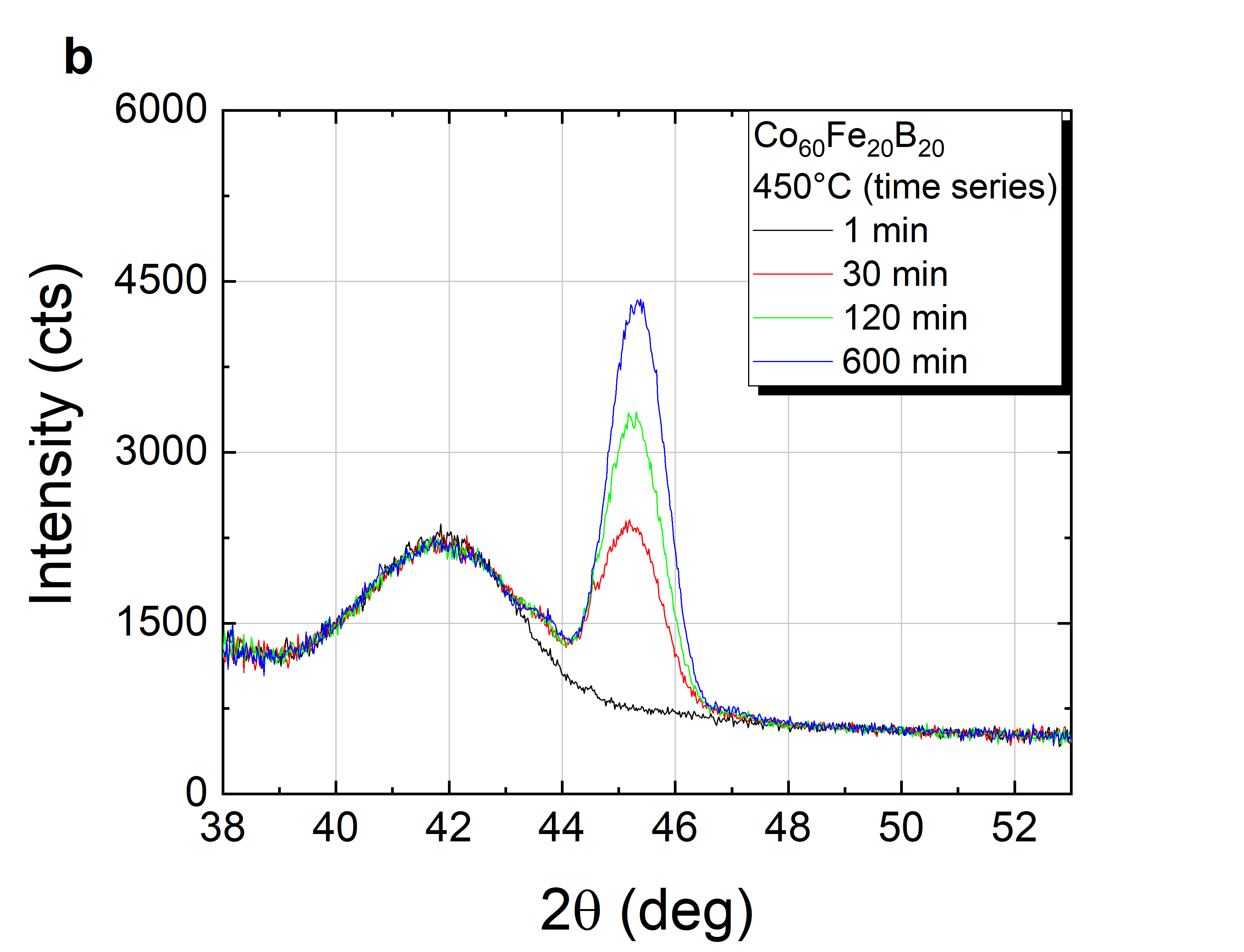 |
| --- | --- |
| **Figure. S1.** XRD *θ*-2*θ* scans of Co_60_Fe_20_B_20_ capped with Ta annealed in oven for 30 min at **(a)** temperatures in the range from 400°C to 600°C and **(b)** at 450°C for different annealing duration. | |

| 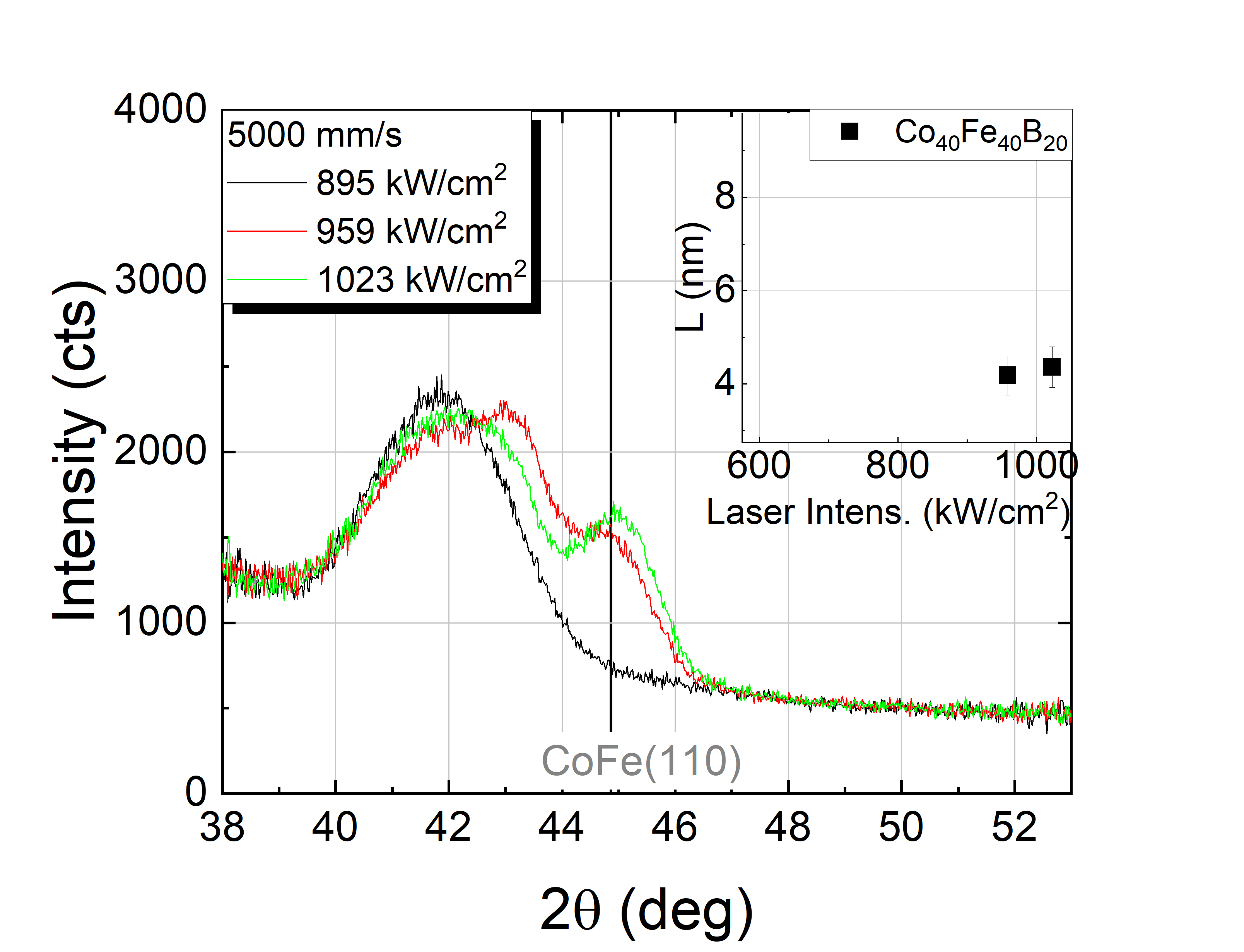 |
| --- |
| **Figure S2.** XRD *θ*-2*θ* scans of Co_40_Fe_40_B_20_ capped with Ta annealed with cw laser radiation at 5000 mm/s. The vertical coherence lengths (*L*) determined from the Co-Fe(110) peak are shown in the inset. |

| 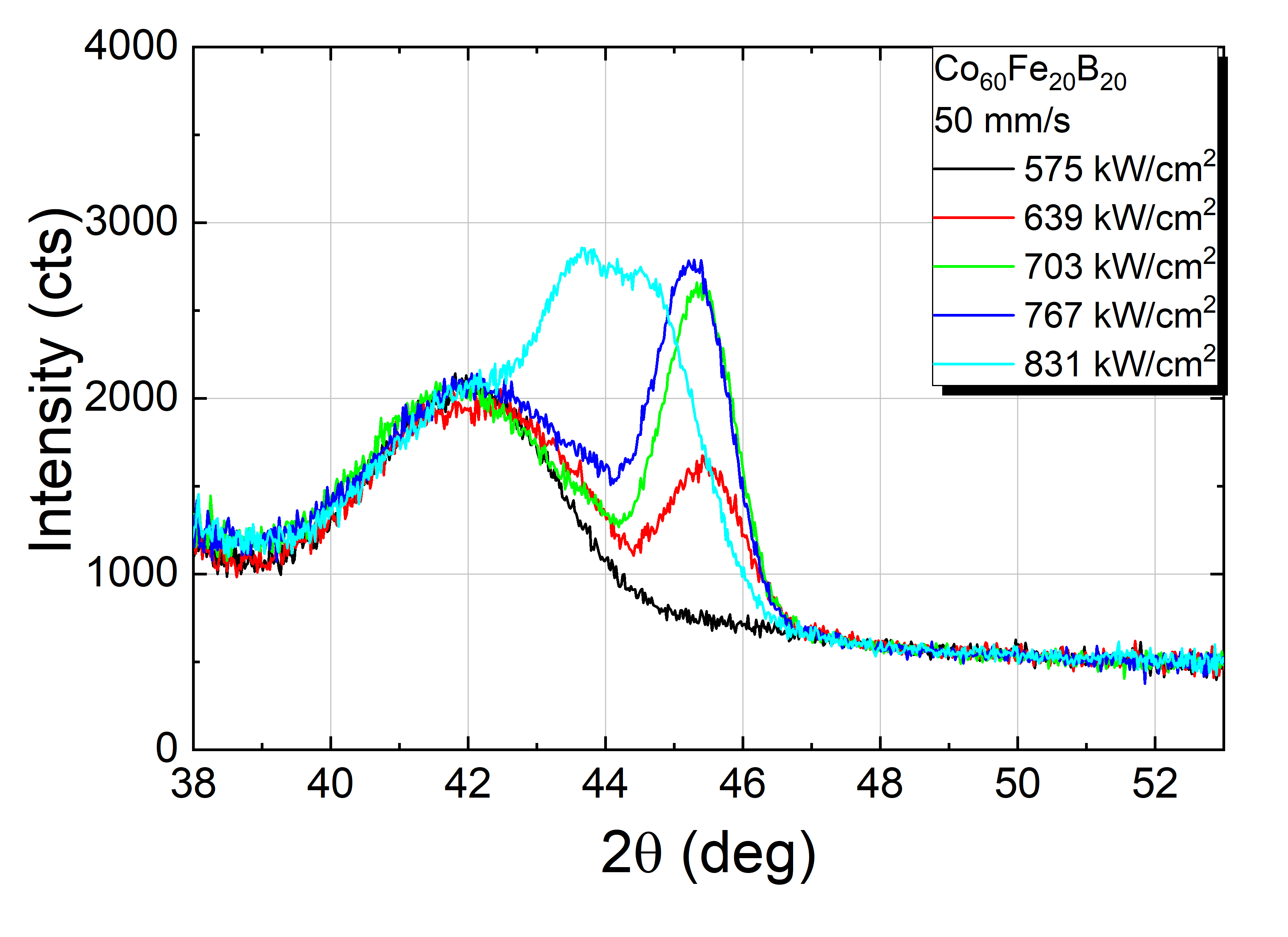 |
| --- |
| **Figure S3.** XRD *θ*-2*θ* scans of Co_60_Fe_20_B_20_ capped with Ta annealed with cw laser radiation at 50 mm/s. |

| 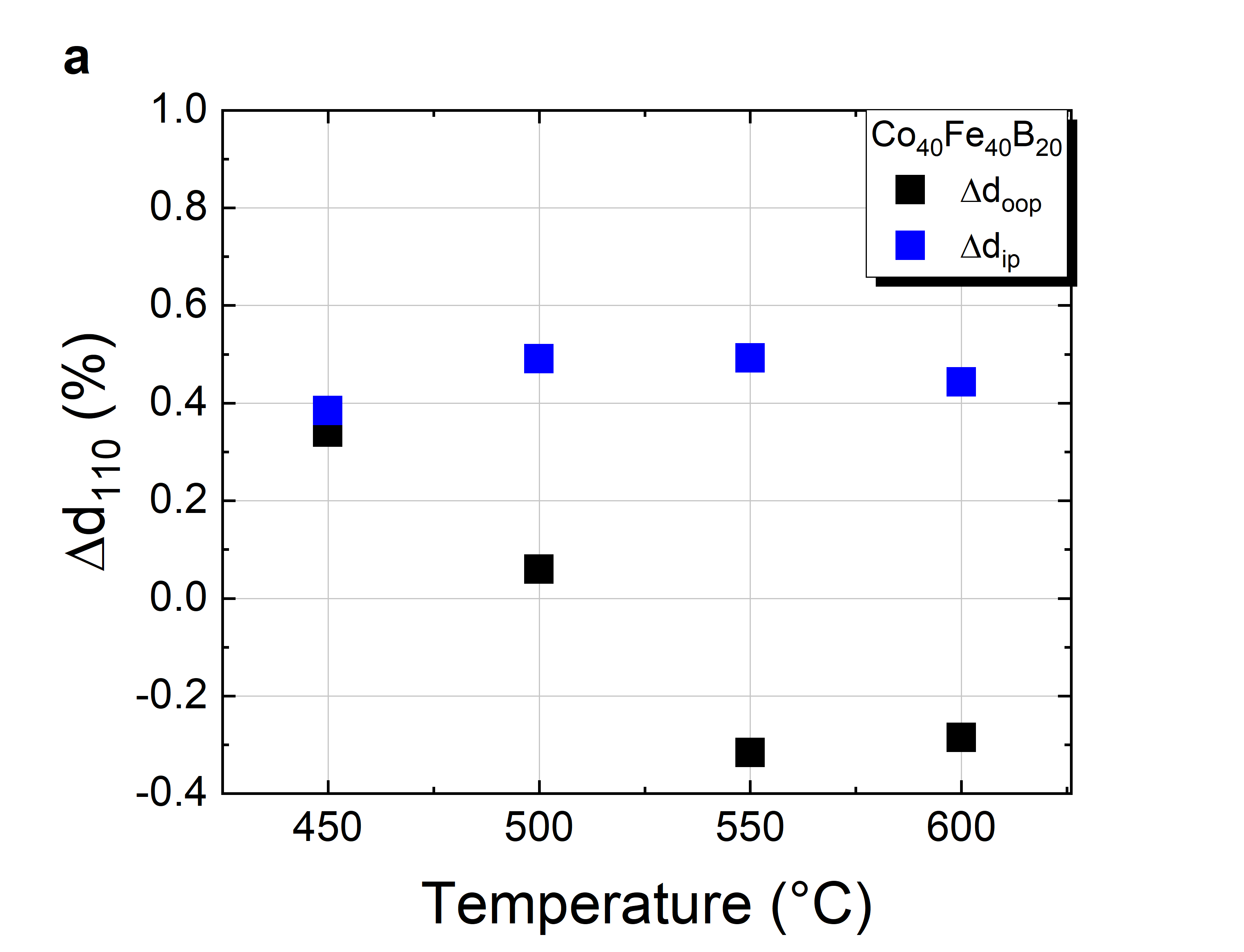 | 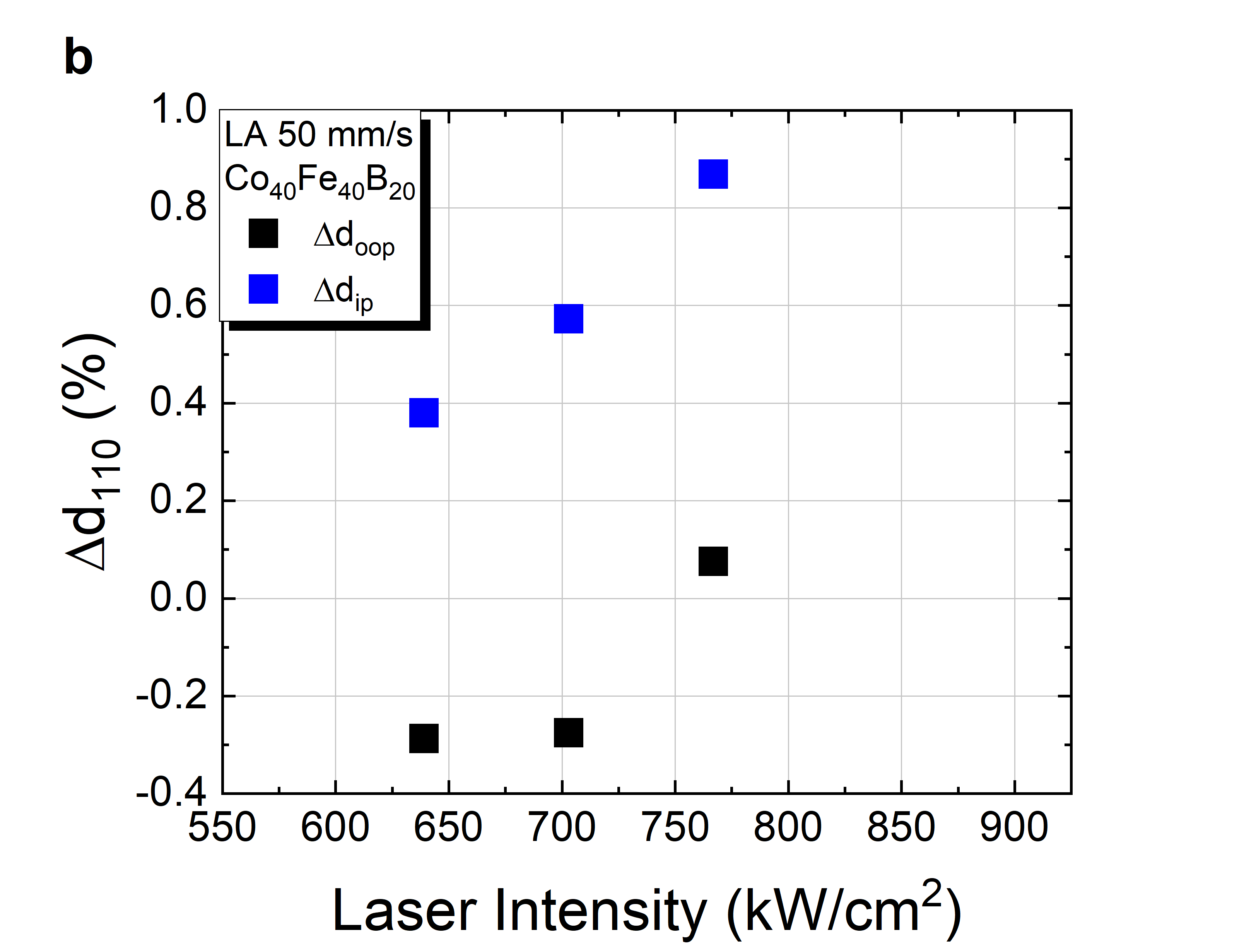 |
| --- | --- |
| **Figure S4.** Depiction of the deviation of *d*-spacing of CoFe(110) planes perpendicular (*d*_ip_) and parallel (*d*_oop_) to the sample surface from database value of Co_40_Fe_40_B_20_ capped with Ta annealed **(a)** in oven 30 min at temperatures of 400°C - 600°C and **(b)** annealed with cw laser at different scanning speed and laser intensity. | |

| 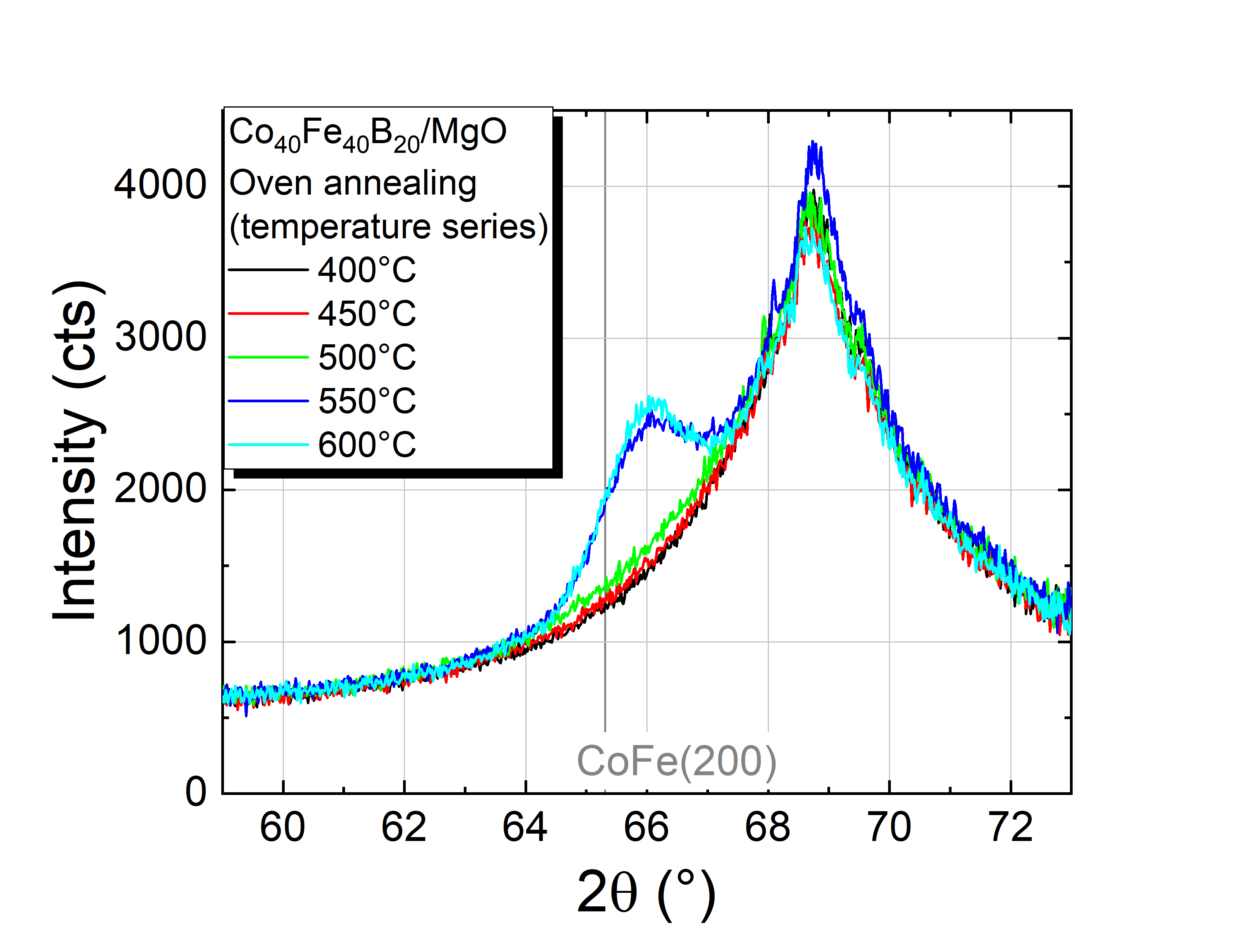 |
| --- |
| **Figure S5.** XRD *θ*-2*θ* scans of Co_40_Fe_40_B_20_ capped with MgO/Ta annealed in oven 30 min at temperatures of 400°C – 600°C. |

| 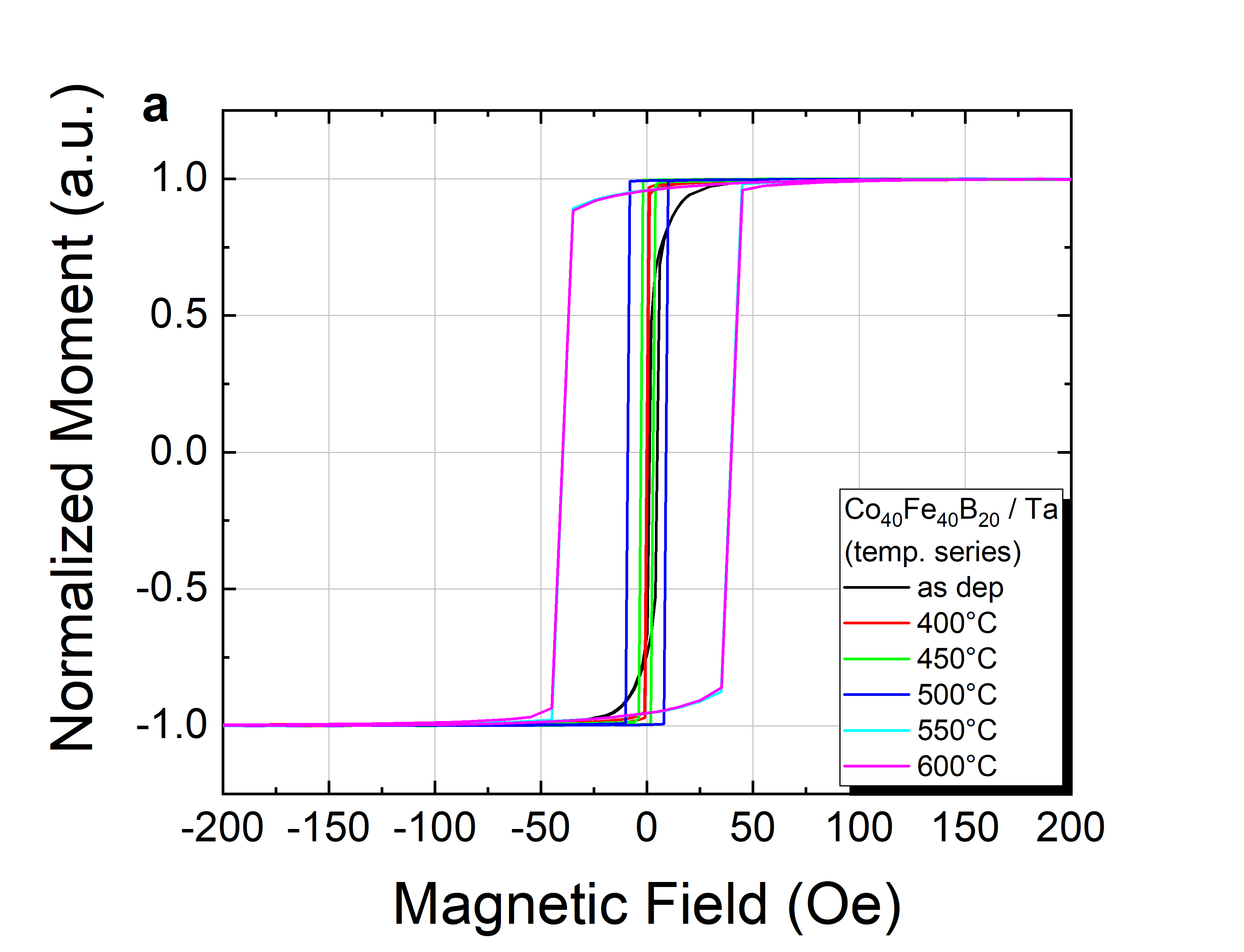 | 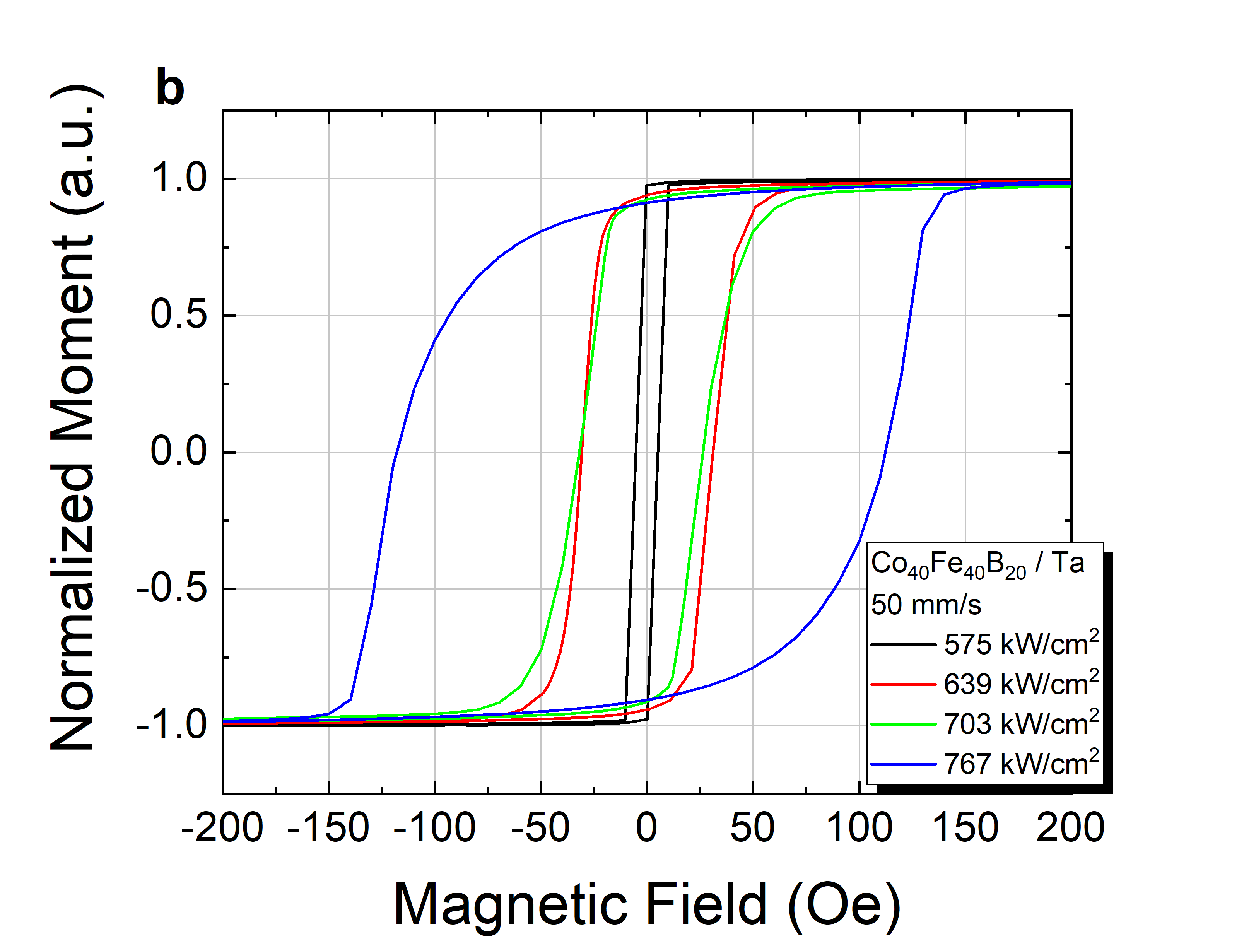 |
| --- | --- |
| **Figure S6.** *M*(*H*) hysteresis loops of Co_40_Fe_40_B_20_ capped with Ta annealed **(a)** in oven 30 min at temperatures of 400°C to 600°C; **(b)** with laser at 50 mm/s scanning speed at different laser intensities. | |

| 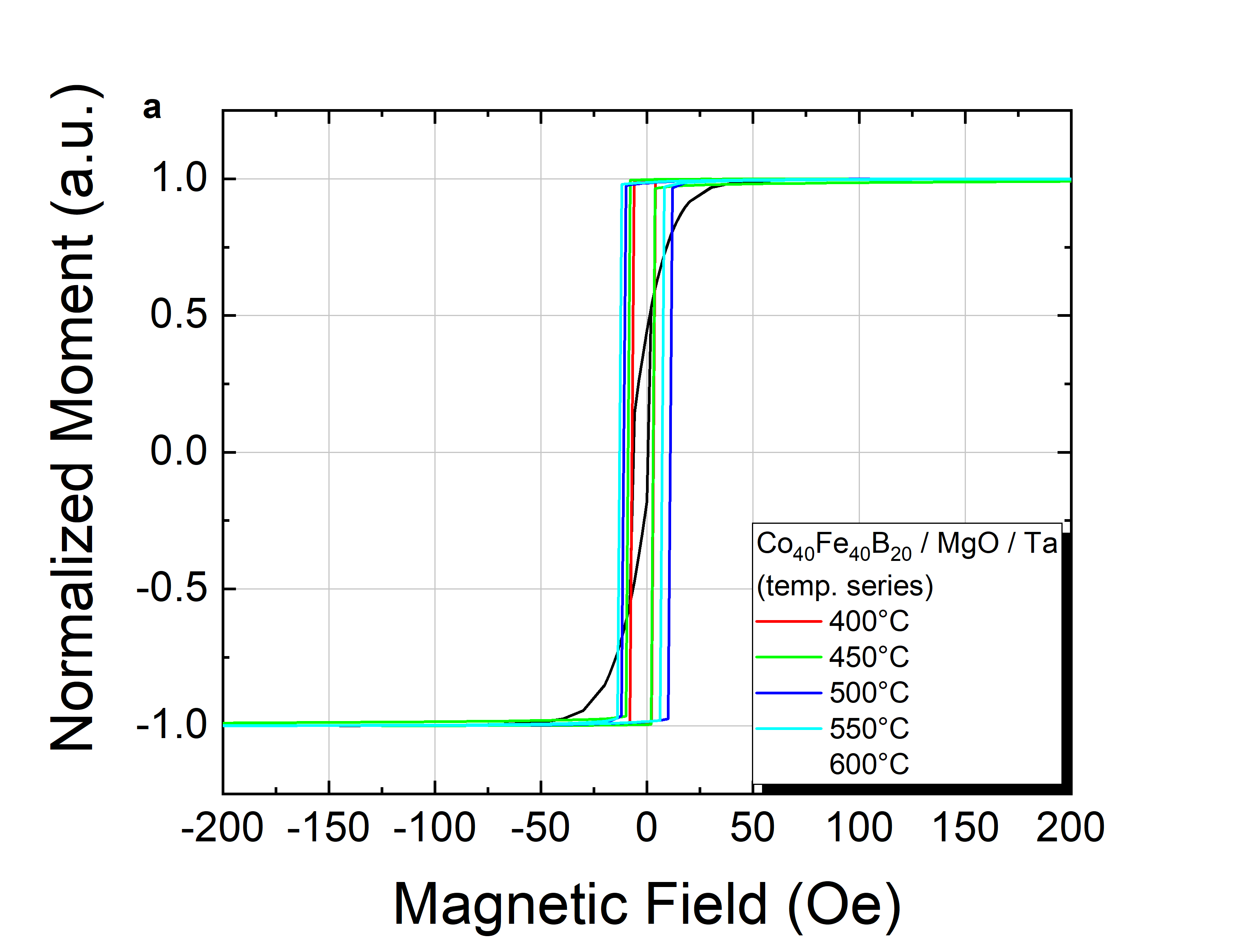 | 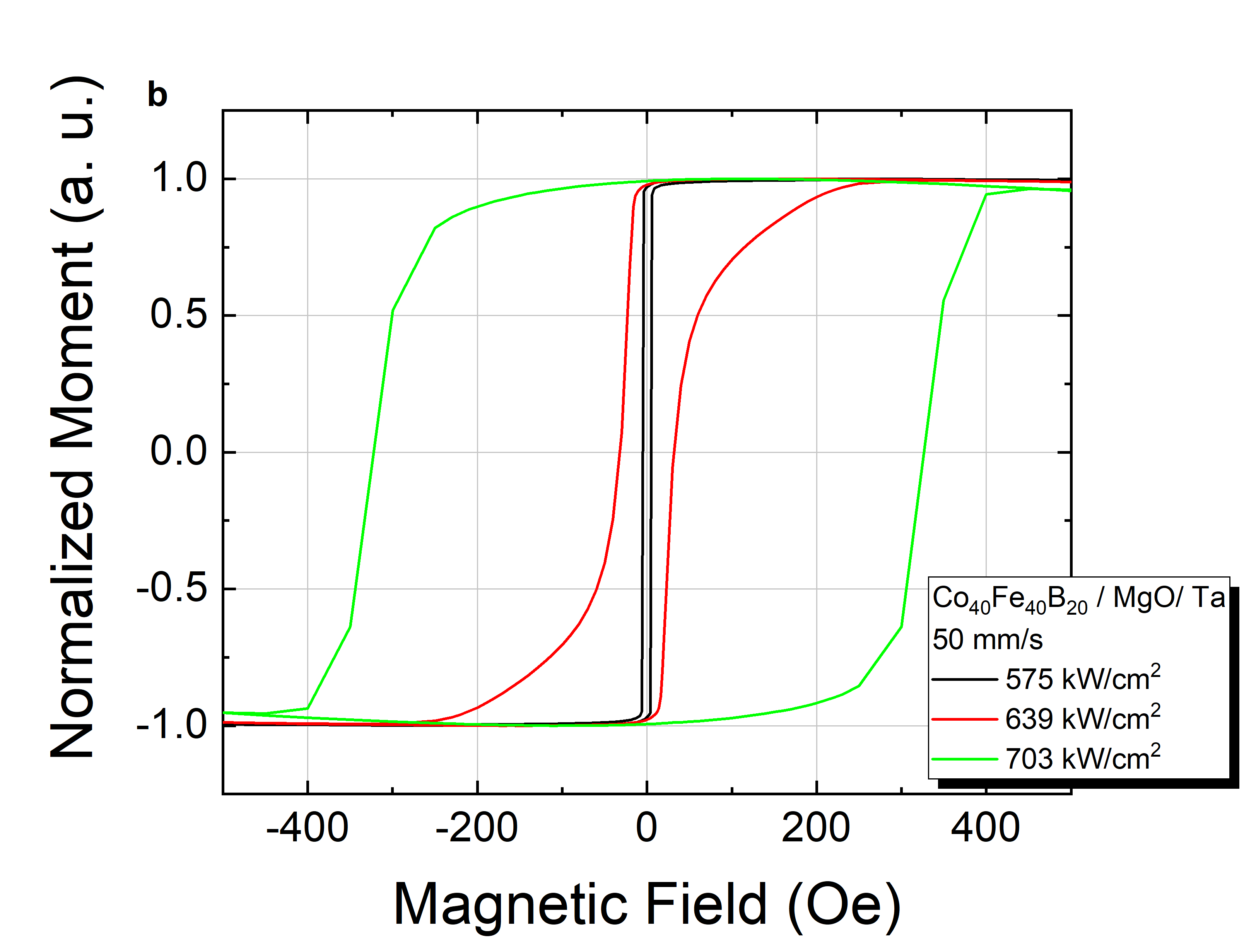 |
| --- | --- |
| **Figure S7.** *M*(*H*) hysteresis loops of Co_40_Fe_40_B_20_ capped with MgO /Ta annealed **(a)** in oven 30 min at temperatures of 400°C to 600°C; **(b)** with laser at 50 mm/s scanning speed at different laser intensities. | |
